# An evolutionary game-theoretic analysis of the "multi-agent co-governance" system of unfair competition on internet platforms

**DOI:** 10.1371/journal.pone.0304445

**Published:** 2024-06-20

**Authors:** Zhen Xu, Shudan Zheng

**Affiliations:** 1 School of Economics and Trade, Chongqing Business Vocational College, Chongqing, China; 2 School of Business Administration, Chongqing Vocational College of Light Industry, Chongqing, China; Southwest University, CHINA

## Abstract

The increasingly prominent issue of unfair competition on Internet platforms (IPUC) severely restricts the healthy and sustainable development of the platform economy. Based on the IPUC "multi-agent co-governance" scenario, this paper introduces stochastic disturbances and continuous strategy set to improve the classical binary deterministic evolutionary game system. The results show that after considering stochastic disturbances, the positive state corresponding to the equilibrium point (1,1) is no longer stable, and the required parameter conditions are more stringent. The IPUC "multi-agent co-governance" system under stochastic disturbances exhibits specific vulnerability. In the continuous strategy set evolutionary game system, government departments and Internet platforms can flexibly make optimal decisions based on maximizing expected returns, and strategy selection has better elasticity. Regardless of the evolutionary game scenario, maintaining the participation level of NGOs and the public above a certain threshold while increasing the penalty intensity is conducive to the evolution of the game system toward the positive state. The analysis process and conclusions provide insights and guidance for the governments to design the IPUC regulatory system and frameworks.

## 1 Introduction

Platform economy, as a typical representative of the new digital economy format and an essential vehicle for digital integration, has played a crucial role in promoting circulation, facilitating smooth economic cycles, enhancing daily life convenience, and ensuring the well-being of the people [1]. Against the challenges faced by China’s economy, the platform economy has shown a remarkable trend of rapid growth, serving as a vital driver for macroeconomic expansion. As the core carriers of the platform economy, Internet platforms have risen in tandem with its rapid growth. The number of Internet platforms in areas such as e-commerce, social networking, and lifestyle and entertainment has increased rapidly, gradually penetrating various fields such as public life, consumption, and work. For instance, video-sharing platforms YouTube and TikTok provide users with digital video viewing and sharing services. Facebook and Twitter have become vital social media platforms for users to share personal updates, browse the news, and disseminate information [2]. E-commerce platforms Amazon and Alibaba provide users with convenient online purchasing services. Internet platforms have fundamentally transformed resource allocation and organizational operational models, significantly elevating the economy’s and society’s digitization level. However, driven by the allure of economic gains, Internet platforms, as profit-oriented market entities, employ their market dominance and technological advantages to engage in unfair competition activities. Various Internet platform competition chaos such as "user privacy theft, price discrimination, forced exclusivity, self-preferential treatment, exclusive monopoly "has gradually emerged [3–5], seriously infringing on the public’s rights and interests, impeding the platform economy’s healthy and orderly development. Typical cases are shown in Table 1. Enhancing the efficiency of unfair competition on Internet platforms (IPUC) regulation while maintaining market order and safeguarding consumer rights has become an urgent and significant challenge facing countries worldwide.

**Table 1:** Typical IPUC cases.

Internet platforms	Time	Details	Result
Amazon	July 2023	Colluding with Apple Inc. to restrict the sale of products from Apple’s competitors.	The Spanish antitrust regulatory authority CNMC imposed a fine of 50.5 million euros on Amazon.
Shein	July 2023	Shein utilizes its dominant market position to coerce clothing manufacturers into signing exclusive agreements, preventing them from collaborating with Temu.	It is currently in the legal litigation phase, with no results yet.
DoorDash	May 2023	Charging additional fees to iPhone users through algorithms.	It is currently in the legal litigation phase, with no results yet.
Ctrip	December 2021	Applying algorithms to impose additional charges on some users.	The defendant, Ctrip, compensated the plaintiff with a threefold difference refund.
Alibaba	November 2020	Merchants are compelled to choose between Alibaba and competing e-commerce platforms.	The Chinese Market Regulatory Authority imposed a fine of 18.228 billion yuan on Alibaba.
Google	June 2017	Abusing dominance as search engine by giving illegal advantage to own comparison shopping service	The European Commission imposed a fine of 2.42 billion euros on Google.

China and the United States have the highest levels of development in the global internet industry, making IPUC regulation strategies a focal point of attention worldwide. In the United States, IPUC regulation follows a "bottom-up" governance philosophy, with Internet platforms at the center. It primarily relies on market mechanisms to self-regulate against unfair competition practices, with platform self-discipline and social supervision playing significant roles [6]. In contrast, China’s IPUC regulation adheres to the "top-down" governance philosophy, with government departments at the center. It mainly employs decisive government intervention through administrative means to curb unfair competition practices [7]. Due to the multifaceted nature of IPUC regulation encompassing political, economic, and social aspects, combined with the inherent complexity of regulating emerging phenomena on Internet platforms, China and the United States continue to face certain limitations in applying their regulatory philosophies and models. With numerous Internet platforms and vast market space, China has seen shortcomings in its government-led regulatory model, such as inadequate regulatory resources, low efficiency, and a lack of flexibility [8]. Consequently, in the face of new platform formats, increasing complexity in market transactions, and growing regulatory demands, the Chinese government has been gradually incorporating the advantages of the U.S. "bottom-up" governance philosophy. It is moving toward a pathway of collaborative regulation involving multiple agents [9]. In summary, IPUC regulation is a complex and systematic large-scale project, and the Chinese government is committed to building an Internet governance system that involves the collaborative participation of NGOs and the public. Therefore, how NGOs and public participation influence the regulatory strategies of governments and competition strategies of internet platforms and their impact on regulatory effectiveness remains a worthwhile research topic. Evolutionary game theory, as a classic dynamic game theory, is a crucial tool for analyzing the strategic choice and interaction of game participants [10–12]. Evolutionary game theory has in-depth applications in prisoner dilemma and public goods games [13, 14].

The structure of this paper is as follows. Section 2 reviews relevant literature, find research deficiencies, and propose research innovations. Section 3 proposes research questions and research hypotheses. Section 4 constructs evolutionary game models under three scenarios and analyzes evolutionary stability and pathways. Section 5 uses case and Matlab to conduct a simulation analysis of the game model. Section 6 draws research conclusions and puts forward research prospects.

## 2 Literature review

### 2.1 The research related to IPUC regulation

The effective regulation of IPUC has sparked extensive discussions in the academic community. Some scholars have taken policy and legislation as their starting point to examine IPUC laws, focusing on dimensions such as behavioral identification [15], scope of application [16], and legal boundaries [17]. Such studies focus on analyzing regulatory policy from a legal perspective and do not address how government departments can enhance regulatory efficiency. As a result, many scholars stand the government-oriented perspective and propose regulatory strategies from a macro-level standpoint. Some of these studies apply the regulatory approach for unfair competition from traditional industries to IPUC and propose strategies based on the "unitary regulation" theory [18]. Aiming at the increasingly prominent P2P platform chaos, Pang et al. [19] explored the critical factors affecting the government’s regulatory strategy based on the data of 18 P2P platforms in Guangdong Province. Hong and Xu [20] took the Alibaba e-commerce platform as a case study object and sorted out the evolution of China’s government-led Internet platform regulatory model. However, due to the rapid evolution and development of internet technology, the effectiveness of the government’s unitary regulation mode has been declining, and its applicability has been diminishing [21]. In recent years, with the continuous deepening and improvement of collaborative governance theory [22, 23], related research has gradually transformed from "unitary regulation" to "multi-agent co-governance." He and Zhang [24] believes that in the face of massive data information and complex market transactions in the Internet platform market, the government faces problems such as tight fiscal budgets and insufficient technical capabilities. It is necessary to rely on organic collaboration among public organizations, businesses, the public, and other social entities to enhance regulatory effectiveness. You [25] systematically reviewed the policies and regulations governing e-commerce in China and developed a collaborative regulatory framework for e-commerce platforms within the context of the new governance model. Finck [26] argued that a polycentric model based on the involvement of stakeholders like the general public is a practical approach to regulating the platform economy. Through comparative analysis, he has substantiated the superiority of collaborative regulation over "top-down" regulation and self-regulation models.

### 2.2 The application of evolutionary game theory in IPUC

Evolutionary games explain the strategic evolution process of selfish individuals [27, 28]. Internet platforms, as market operators, have the characteristics of "economic agents." Driven by economic interests, Internet platforms obtain illegal profits through unfair competition methods such as price discrimination and privacy theft. The government, as the representative of public authority, is the core subject in regulating IPUC. In the "multi-agent co-governance" scenario, the public, consumers, and platform users are critical supplements to IPUC regulation. Therefore, existing research primarily utilizes evolutionary game theory to study the strategic interaction mechanism among governments, internet platforms, the public, and users.

While big data technology improves the market competitiveness of enterprises, it has also become a crucial tool to implement price discrimination. Chai and Wang [29] combined evolutionary game theory and the Lotka-Volterra model to explore the influence of government regulatory behavior on enterprises’ price discrimination decisions. The study shows that government measures such as public awareness campaigns, technological incentives, and punitive taxation can effectively curb enterprise price discrimination. Li et al. [30] applied evolutionary game theory and numerical simulation to analyze the strategic evolutionary paths of governments, enterprises, and consumers. The results indicate that government regulation, consumer complaints, and media exposure can effectively reduce the probability of enterprises implementing price discrimination. The consumers can only "vote with their feet" when the governments relax regulation, making it challenging to protect individual rights and interests. Wu et al. [31] constructed an evolutionary game model based on PT-MA theory from the perspective of government-consumer cooperation. He pointed out that governments can promote e-commerce platforms to set reasonable product prices by increasing incentives for consumer participation and penalties for price discrimination.

In the Internet era, data sharing and trading have made data a valuable asset, leading to concerns about data privacy breaches. Wang et al. [32] verified the effectiveness of dual regulation by the governments and users by constructing an evolutionary game model involving governments, digital platforms, and users, thereby preventing digital platforms from abusing user data and protecting user privacy. Gu et al. [33] took internet platforms and users as game participants and used MATLAB to analyze the evolutionary paths of both sides’ strategies. It is found that information leakage loss and information sensitivity are critical factors affecting users’ provision of digital information. Government regulation is conducive to guiding Internet platforms to use user information correctly and avoid user privacy leakage. Overall, evolutionary game theory has been preliminarily applied in the IPUC field, laying a theoretical foundation for this paper.

### 2.3 The shortcomings of existing research and innovation of this paper

It is worth noting that there are two areas for improvement in the research conducted by existing scholars when applying evolutionary game theory. Firstly, the changing law of the game agents’ strategic choice probability over time is written as a deterministic formula. However, strategic choices frequently change due to external stochastic factors such as policy adjustments, market fluctuations, and technological innovations. Deterministic evolutionary games struggle to accurately capture the ongoing effect of numerous stochastic factors on dynamic systems in the real world. Secondly, the game agents’ strategic choices are set as "binary" or "ternary" sets, whereas in reality, the strategic choices often exhibit continuous and gradual characteristics. The discrete strategy set struggles to objectively portray the gradual nature of game agents’ strategic choices. The abovementioned shortcomings reduce the alignment between the model and reality, decreasing the congruence between research findings and IPUC regulatory practices.

Compared with existing research, this paper has the following innovations. (1)We introduce stochastic disturbances and continuous strategy set into the evolutionary game model for IPUC regulation, addressing the shortcomings in existing research regarding model construction. (2) We progressively deepen the characterization of the real-world context of IPUC regulation, introduce NGOs and the public as collaborative regulatory agents into the game model, and use case analysis and MATLAB tools to explore the evolutionary dynamics of the game system under “multi-agent co-governance” system.

This paper makes contributions in three aspects. Firstly, the classical evolutionary game model is extended and improved. We analyzed the evolutionary pathways and results of the game system after introducing stochastic disturbances and continuous strategy set. Secondly, We consider the participation of NGOs and the public, and the decision-making interaction mechanisms between government departments and internet platforms are explored in three different game scenarios. Finally, the MATLAB tool is applied to simulate the evolutionary pathways, equilibrium points, and parameter sensitivity of the game system.

## 3 Problem description and model assumptions

### 3.1 Problem description

**Fig 1:**
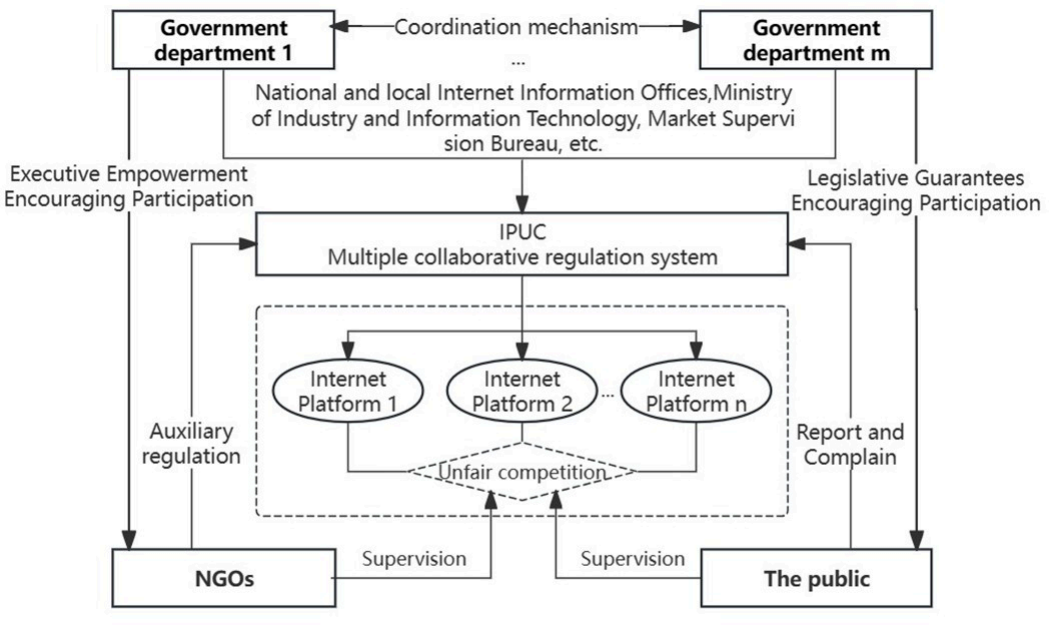
The IPUC "multi-agent co-governance" system.

Compared with unfair competition practices in traditional industries, those emerging in the internet landscape exhibit a high degree of dynamism, complexity, and stealthiness, reducing the applicability of the traditional "unitary regulation" model. This paper considers the collaborative role of NGOs and the public and constructs a IPUC "multi-agent co-governance" system, as illustrated in Fig 1.

The "multi-agent co-governance" system utilizes industry associations and platform users as supplementary third-party forces to assist government departments in regulating Internet platforms. The collaboration of multiple agents is manifested in two aspects. On the one hand, government departments formulate regulatory policies and use their regulatory authority to restrain unfair competition practices by internet platform market entities. Standard administrative enforcement measures include fines, public notices, and suspension of business for rectification [34]. For example, on October 8, 2021, the China State Market Supervision Bureau imposed administrative penalties on the Meituan Delivery Platform for its forced exclusivity monopolistic behavior by the Anti-Monopoly Law and the Anti-Unfair Competition Law, resulting in a total fine of 3.442 billion yuan [35]. On the other hand, the collaborative entities in IPUC regulation include Non-Governmental Organizations (NGOs) [36] primarily composed of industry associations and social groups, as well as the public [37] primarily composed of merchants and consumers. The NGOs leverage their organizational and human resources to assist government departments in conducting investigations, gathering evidence, and carrying out administrative enforcement actions [38]. For example, in November 2021, the China Guangzhou Market Supervision Bureau collaborated with e-commerce NGOs to launch the "Internet Sword Action," which focused on cracking down on unfair competition practices like price discrimination and counterfeit sales on e-commerce platforms. Simultaneously, the public plays a significant role in social supervision by providing information to government departments through phones and the Internet [39]. For example, in August 2018, the China Shanghai government investigated allegations of consumer privacy infringement against the social commerce platform "Red Little Book" based on consumer complaints and reports, ultimately fined it 50,000 yuan. The Chinese government has explicitly incorporated the roles of NGOs and the public in internet platform regulation into policy and legal documents. For instance, the policy "Several Opinions on Promoting the Normative and Healthy Development of the Platform Economy" explicitly states: Strengthen social supervision and explore the regulatory mechanisms for the platform economy with the participation of the public and third-party professional organizations.

As the regulation leader, government departments aim to maintain market competition order, enhance social welfare, improve performance, and build public trust. They are responsible for promulgating and refining regulatory policies utilizing legal systems and supporting law enforcement means to regulate the competitive activities of Internet platforms. As profit-driven entities in the market, Internet platforms aim to increase market share, revenue, and profits. They are susceptible to engaging in unfair competition practices driven by economic interests. As independent social organizations, NGOs aim to alleviate the regulatory burden on the government and enhance regulatory efficiency. As direct victims of IPUC, the public is in a relatively disadvantaged position in the regulatory system, relying on government administrative enforcement to protect their rights and interests.

In summary, inherent conflicts and game relationships are inevitable, given the significant differences in these stakeholders’ roles, objectives, and interests. Government departments and Internet platforms are set as direct game agents to facilitate model construction and analysis. NGOs and the public are introduced into the game framework as indirect participants. All parties exhibit bounded rationality characteristics and continuously adjust their strategic choices based on expected benefits.

### 3.2 Model assumptions

Based on the method of evolutionary games, this paper analyzes the IPUC regulatory system under the binary strategy set and the continuous strategy set, studies the strategic interaction mechanism between government departments and Internet platforms. The meaning of critical parameters is shown in Table 2.

**Table 2:** The meaning and value range of critical parameters.

Symbol	Parameter meaning	Value range
*S*	Regulatory benefits	*S* > 0
*c*_0_	Fixed regulatory cost	*S* > *c*_0_ > 0
*p*	NGO participation	0 ≤ *p* ≤ 1
*e*	NGO power space	0 ≤ *e* ≤ 1
*α*	Regulatory ability	*a* > 0
*m*	Regulatory intensity	0 ≤ *m* ≤ 1
*b*	Regulatory cost coefficient	*b* > 0
*w*	Increased social welfare	*w* > 0
*v*	Loss of social welfare	*v* > 0
*r*	Reputational loss	*r* > 1
*q*	Public participation	0 ≤ *q* ≤ 1
*H*	Essential benefits	*H* > 0
Δ*h*	Gains from unfair competition	Δ*h* > 0
*β*	Capability of unfair competition	*Β* > 0
*n*	Unfair competition level	0 ≤ *n* ≤ 1
*d*	Unfair competition cost coefficient	*d* > 0
*g*	Penalty intensity	0 ≤ *g* ≤ 1
*φ*	Constant	*φ* > 0

**Assumption 1:** The regulatory benefits of government departments are denoted as *S*, including enhanced governmental performance, improved public trust, and increased tax revenue resulting from regulating the platform economy. The regulatory cost includes the human, material, and financial resources invested in policy formulation, administrative supervision, and enforcement penalties. It is expressed as $C=c_{0}+\frac{b}{2\alpha }m^{2}$, composed of fixed cost *C*_0_ and variable cost $\frac{b}{2\alpha }m^{2}$ [40]. Among them, *m* represents the regulatory intensity, *α* represents the regulatory ability, and b represents the regulatory cost coefficient. A higher *α* corresponds to a more robust regulatory capacity, while a more minor *b* results in lower marginal regulatory cost, and the corresponding regulatory cost is also lower. The increase in social welfare due to fair competition in Internet platforms is represented as *w*, while the social welfare loss caused by unfair competition is denoted as *v*. Social welfare includes economic growth, consumer surplus, and improvement of the business environment.

**Assumption 2:** The basic benefits of Internet platforms are denoted as *H*, and the level of fair competition is represented by *n*. Therefore, the additional benefits generated from unfair competition are (1 − *n*)Δ*h*, such as gaining additional market share by suppressing competitors through forced exclusivity behaviors [41], gaining additional profits by practicing price discrimination to raise product and service prices [42], or profiting from selling user privacy [43]. The cost incurred by Internet platforms due to unfair competition is expressed as $\frac{d}{2\beta }{\left(1-n\right)}^{2}$, following the principle of increasing marginal cost. Among them, *β* represents the capability for unfair competition, and *d* represents the cost coefficient. Due to information asymmetry, the probability of being investigated and penalized by government departments is positively correlated with regulatory intensity when Internet platforms adopt unfair competition strategies. To simplify the analysis, designate this probability as *m*, equivalent to regulatory intensity. After being investigated, the penalty for Internet platforms is *gφ* [44], including fines, loss of social reputation, suspension of business for rectification, etc. Among them, *g* represents the penalty intensity, and *φ* is a constant.

**Assumption 3:** The NGOs exhibit both synergy and substitution with government regulation, allowing them to partially share the regulatory cost with the government and effectively alleviate the shortage of administrative regulatory resources [45]. For example, they can assist the government in investigations, evidence gathering, information collection, and analysis. The government can also delegate some of its work (industry norms, industry standards, special inspections, and rectifications) to NGOs for collaborative implementation. Simultaneously, government departments limit the scope of NGOs’ authority to prevent "power overreach." Therefore, the regulatory cost [46] is rewritten as $C=c_{0}+\frac{b\left(1-ep\right)}{2\alpha }m^{2}$. In this equation, the NGO participation level is denoted as *p*, and power space is represented by *e*. This implies that the variable regulatory cost decreases as p and e increase. As direct victims of IPUC, the public can report and complain to government departments through multiple channels [47]. If government departments fail to take action, they will incur losses denoted as *qr*. Among them, *r* represents reputation loss, and *q* represents public participation, which reflects the public’s awareness of rights safeguard, tolerance, and resistance to IPUC.

## 4 Evolutionary mechanism analysis

The strategy set of government departments is (active regulation, passive regulation), corresponding to the intensity of regulation *m* = (1,0). The probability of adopting active regulation strategy is denoted as *x*(0 ≤ *x* ≤ 1), and the probability of adopting passive regulation strategy is 1 − *x*. The strategy set of Internet platforms is (fair competition, unfair competition), corresponding to the intensity of fair competition *n* = (1,0). The probability of adopting fair competition strategy is denoted as *y*(0 ≤ *y* ≤ 1), and the probability of adopting unfair competition strategy is 1 − *y*. Since NGO participation, public participation, and penalty intensity are changeable parameters, while other parameters remain relatively stable over a short period, they are not easily altered. Therefore, this paper focuses on analyzing how to adjust the critical parameters *p*,*g*, and *q* values to guide the game system toward the positive state (1,1).

### 4.1 Binary strategy set evolutionary game

#### 4.1.1 Binary deterministic evolutionary game

Substituting *m* = (0,1), *n* = (0,1) into the model assumptions, the strategic payoff matrix of the binary deterministic evolutionary game can be obtained as Table 3.

**Table 3:** The strategic payoff matrix of government departments and Internet platforms.

Game agents		Internet platform	
		Fair competition *y*	Unfair competition 1 − *y*
**Government department**	Active regulation *x*	$\left(S-c_{0}-\frac{b(1-ep)}{2\alpha }+w,H\right)$	$\left[S-c_{0}-\frac{b\left(1-ep\right)}{2\alpha }-v,H+\Delta h-\frac{d}{2\beta }-\varphi g\right]$
	Passive regulation 1 − *x*	(*w*,*H*)	$\left(-v-qr,H+\Delta h-\frac{d}{2\beta }\right)$

The expected benefits obtained by government departments choosing the "active regulation" and "passive regulation" strategies are:

$$E_{x}=y[S-c_{0}-\frac{b\left(1-ep\right)}{2\alpha }+w]+(1-y)[S-c_{0}-\frac{b\left(1-ep\right)}{2\alpha }-v],$$ (1)

$$E_{1-x}=yw+(1-y)(-v-qr).$$ (2)

The expected benefits obtained by Internet platforms choosing the "fair competition" and "unfair competition" strategies are:

$$E_{y}=xH+(1-x)H,$$ (3)

$$E_{1-y}=x(H+\Delta h-\frac{d}{2\beta }-\varphi g)+(1-x)(H+\Delta h-\frac{d}{2\beta }).$$ (4)

According to Eqs (1)–(4), the replication dynamic equations of government departments and Internet platforms are obtained:

$$F\left(x\right)=\frac{dx}{dt}=x\left(1-x\right)\left\{\left[S-c_{0}-\frac{b\left(1-ep\right)}{2\alpha }+qr\right]-qry\right\},$$ (5)

$$F\left(y\right)=\frac{dy}{dt}=y\left(1-y\right)\left[\left(-\Delta h+\frac{d}{2\beta }\right)+\varphi gx\right].$$ (6)

**Proposition 1:** Under the binary strategy set, the positive state (1,1) is the evolutionary stable strategy (ESS) of the deterministic game system. It must satisfy the following threshold conditions:

$$p>\frac{b-2\alpha (S-c_{0})}{be},g>\frac{2\beta \Delta h-d}{2\beta \varphi }.$$ (7)

**Proof:** The replication dynamic Eqs (5) and (6) demonstrate the evolutionary process of strategic choices for government departments and Internet platforms. Let *F*(*x*) = *F*(*y*) = 0, we can get that there are five equilibrium points in the game system, namely (0,0), (0,1), (1,0), (1,1), (*x*_0_,*y*_0_). Among them, $x_{0}=\frac{2\beta \Delta h-d}{2\beta \varphi g},y_{0}=\frac{2\alpha (S-c_{0}+qr)-b\left(1-ep\right)}{2\alpha qr}$. According to the method proposed by Friedman [48], when the Jacobian matrix of the equilibrium point satisfies *Tr J* < 0, *Det J* > 0, the equilibrium point is the ESS of the game system. The trace of the Jacobian matrix of the equilibrium point (*x*_0_,*y*_0_), *Tr J* = 0, does not satisfy the condition for ESS. For the "multi-agent co-governance" system of IPUC, the equilibrium point (1,1) represents the ultimate goal the system needs to achieve. Therefore, we focus on analyzing the parameter conditions and evolutionary pathways of equilibrium point (1,1).

The Jacobian matrix corresponding to the equilibrium point (1,1) is as follows:

$$J=\left[\begin{array}{cc} -\left[S-c_{0}-\frac{b\left(1-ep\right)}{2\alpha }\right] & 0\\ 0 & -\left[(-\Delta h+\frac{d}{2\beta })+\varphi g\right] \end{array}\right].$$ (8)

It can be obtained:

$$\left[S-c_{0}-\frac{b\left(1-ep\right)}{2\alpha }\right]>0,\left[\left(-\Delta h+\frac{d}{2\beta }\right)+\varphi g\right]>0.$$ (9)

Deformed to obtain Eq (7), Proposition 1 is proved.

Proposition 1 suggests that in the practice of IPUC regulation, government departments and Internet platforms find it challenging to accurately observe each other’s strategic choices due to information asymmetry. Accordingly, in the initial stages of the game, they exhibit various strategy combinations, and through repeated interactions, they evolve toward different equilibrium points. The NGO participation and penalty mechanism are crucial in guiding the system toward the positive state. Maintaining parameters *p* and *g* above certain thresholds effectively regulates IPUC and promotes the healthy and orderly development of the platform economy.

#### 4.1.2 Binary stochastic evolutionary game

While deterministic evolutionary games capture the dynamic nature of games, they primarily focus on the deterministic decision-making of game agents and do not consider the effect of stochastic disturbances on their strategic choices. However, in the practice of IPUC regulation, game agents exhibit bounded rationality, and their strategic choices are highly affected by internal factors such as speculative behavior and moral risks, as well as external stochastic factors like policy adjustments and market fluctuations. Scholars have undertaken practical explorations to address the effect of external stochastic disturbances and continuous strategy sets on game systems. The Gaussian white noise interference term has been introduced into the replication dynamic equation to analyze the evolutionary pathways of strategy in a stochastic evolutionary game model [49]. For instance, Shan et al. [50] has developed a stochastic evolutionary game model for urban public crisis management, considering the high uncertainty and dynamics of public crisis events by introducing the Gaussian white noise disturbance. Li et al. [51] created a stochastic evolutionary game model among governments, enterprises, and consumers to study the strategic interaction mechanisms in the construction of charging infrastructure.

Therefore, this paper introduces Gaussian white noise stochastic disturbances to improve the replication dynamic equations of the game system, thereby improving the degree of fit between the model results and reality. Since 1 − *x*(*t*) ≥ 0 does not affect the analysis results, the replication dynamic Eqs (5) and (6) are rewritten as:

$$F\left(x\right)=x(t)\left\{\left[S-c_{0}-\frac{b\left(1-ep\right)}{2\alpha }+qr\right]-qry(t)\right\}+x(t)\sigma d\omega (t),$$ (10)

$$F\left(y\right)=y\left(t\right)\left[\left(-\Delta h+\frac{d}{2\beta })+\varphi gx(t\right)\right]+y(t)\sigma d\omega (t),$$ (11)

where *σ* is stochastic disturbance intensity, *ω*(*t*) is a one-dimensional standard Brownian motion, reflecting the interference of external stochastic factors on the game system. d*ω*(*t*) represents Gaussian white noise, and when *t* > 0 with a step size *h* > 0, its increment Δ*ω*(*t*) = *ω*(*t* + *h*) − *ω*(*t*) follows a normal distribution $N(0,\sqrt{h})$. Assuming at the initial moment *t* = 0, *x*(0) = *y*(0) = 0, it can be deduced that d*ω*(*t*)|_*t* = 0_ = 0, indicating that the equation has at least zero solution, that is, the solution to the initial value problem of the differential equation. Without white noise interference, the system will remain in this state indefinitely. We refer to the solutions of literature [52, 53] and judge the stability of the game system based on the stochastic differential equation theorem.

**Theorem 1:** Suppose the stochastic process *X* = {*X*(*t*), *t* ≥ 0} is the solution of the differential equation dXt=ft,Xtdt+g(t,X(t))dω(t)Xt0=x0, where there exists a continuously differentiable function *V*(*t*,*x*) and positive constants *c*_1_ and *c*_2_ such that *c*_1_ |*x*|^*k*^ ≤ *V*(*t*,*x*) ≤ *c*_2_ |*x*|^*k*^. In this case, if there exists a positive constant *γ* such that *LV*(*t*,*x*) ≤ − *γV*(*t*,*x*), then the zero solution to this initial value problem of the differential equation is k-order moment exponent stable. If there exists a positive constant *γ* such that *LV*(*t*,*x*) ≥ *γV*(*t*,*x*), then the Non-zero solution to this initial value problem of the differential equation is k-order moment exponent unstable. Here, $LV\left(t,x(t)\right)=V_{t}\left(t,x\right)+V_{x}\left(t,x\right)f\left(t,x\left(t\right)\right)+\frac{1}{2}g^{2}(t,x)V_{xx}\left(t,x\right)$.

Regarding Eq (10), taking *V*(*t*,*x*(*t*)) = *x*(*t*), *c*_1_ = *c*_2_ = 1, *k* = 1, *γ* = 1, then *LV*(*t*,*x*(*t*)) = *f*(*t*,*x*(*t*)). When the initial value problem’s solution is k-order moment exponent stable, *f*(*t*,*x*(*t*)) ≤ − *x*(*t*), which implies $\left[S-c_{0}-\frac{b\left(1-ep\right)}{2\alpha }\right]+1\leq qr[y\left(t\right)-1]$. Since 0 ≤ *y*(*t*) ≤ 1, the parameter condition is $\left[S-c_{0}-\frac{b\left(1-ep\right)}{2\alpha }\right]+1\leq -qr$. In this case, *x*(*t*) = 0 is the stable solution of the equation. The probability of government departments choosing active regulation decreases over time, and the strategic choice eventually evolves toward passive regulation. When the initial value problem’s solution is k-order moment exponent unstable, *f*(*t*,*x*(*t*)) ≥ *x*(*t*), which implies $\left[S-c_{0}-\frac{b\left(1-ep\right)}{2\alpha }\right]-1\geq qr[y\left(t\right)-1]$. Since 0 ≤ *y*(*t*) ≤ 1, the parameter condition is $\left[S-c_{0}-\frac{b\left(1-ep\right)}{2\alpha }\right]-1\geq 0$. In this case, *x*(*t*) = 0 is the unstable solution of the equation. With an increase in the number of games, the probability of the government departments choosing active regulation increases, and the strategic choice eventually evolves toward active regulation. Similarly, regarding Eq (11), the conditions for the initial value problem’s solution to have k-order moment exponent stability and instability are: $(-\Delta h+\frac{d}{2\beta })+\varphi g+1\leq 0,(-\Delta h+\frac{d}{2\beta })-1\geq 0$.

As a result, by modifying the above expression, the conditions required for the game system to evolve toward (1,1) under stochastic disturbances are:

$$\left[S-c_{0}-\frac{b\left(1-ep\right)}{2\alpha }\right]-1>0,(-\Delta h+\frac{d}{2\beta })-1>0.$$ (12)

In the same way, the conditions required for the game system to evolve toward (0,0) under stochastic disturbances are:

$$\left[S-c_{0}-\frac{b\left(1-ep\right)}{2\alpha }\right]+1\leq -qr,\left(-\Delta h+\frac{d}{2\beta }\right)+\varphi g+1\leq 0.$$ (13)

According to Eq (7), in the binary deterministic evolutionary game system, the parameter *g* is a necessary condition for the system to evolve toward the positive state. This indicates that the penalty mechanism has formed an effective deterrent to the Internet platform, and increasing the penalty intensity will help lock the game system into the positive state. Combining Theorem 1 and Eq (12), in the binary stochastic evolutionary game system, the parameter *g* is not reflected in the threshold conditions. This implies that the positive guiding role of the penalty mechanism is limited, and increasing the penalty intensity cannot promote the ESS of the system to lock into the positive state. Nonetheless, when *y*(*t*) deviates from 1, the penalty mechanism will exhibit a reverse guiding effect, hindering the system from evolving into the negative state.

**Proposition 2:** Stochastic disturbances significantly affect game agents’ strategic choices, hindering the game system’s evolutionary process toward the positive state (1,1) and causing a change in the evolutionary pathway.

**Proof:** Comparing Eqs (12) and (9), it is evident that considering stochastic disturbances leads to changes in the parameter threshold conditions required to lock the game system’s ESS into the positive state. Regarding the parameters related to *x*(*t*), the threshold values for *S*, *e*, *p*, and *α* increase, while the threshold values for *b* and *c*_0_ decrease, which implies a higher NGO participation level, more robust regulatory capacity, and lower regulatory cost. Regarding the parameters related to *y(t)*, the threshold value for *d* increases while the threshold values for Δ*h* and *β* decrease, which implies higher unfair competition costs, lower unfair competition benefits, and weaker unfair competition capabilities. Proposition 2 is proved.

Proposition 2 indicates that the applicability of research conclusions in the deterministic game system decreases due to the interference of internal and external stochastic factors. The game relationship between government departments and Internet platforms becomes more complex, increasing uncertainty in the evolutionary dynamic system and raising the challenges of IPUC regulation. On the one hand, increasing the NGO participation level, improving regulatory methods, and reducing regulatory costs can help mitigate the negative effect of stochastic disturbances on the game system. This, in turn, enhances the probability of the system evolving toward the positive state. On the other hand, the effectiveness of the penalty mechanism is significantly affected by stochastic disturbances. While refining the penalty mechanism, government departments should also entirely play the self-regulating role of the market mechanism, encourage self-discipline management of Internet platforms, and create a healthy industry competitive environment.

**Proposition 3:** Under stochastic disturbances, an unstable, chaotic region exists within the game system. When the initial point of the game falls within this region, the game system will evolve toward various equilibrium points after multiple repeated games.

**Fig 2:**
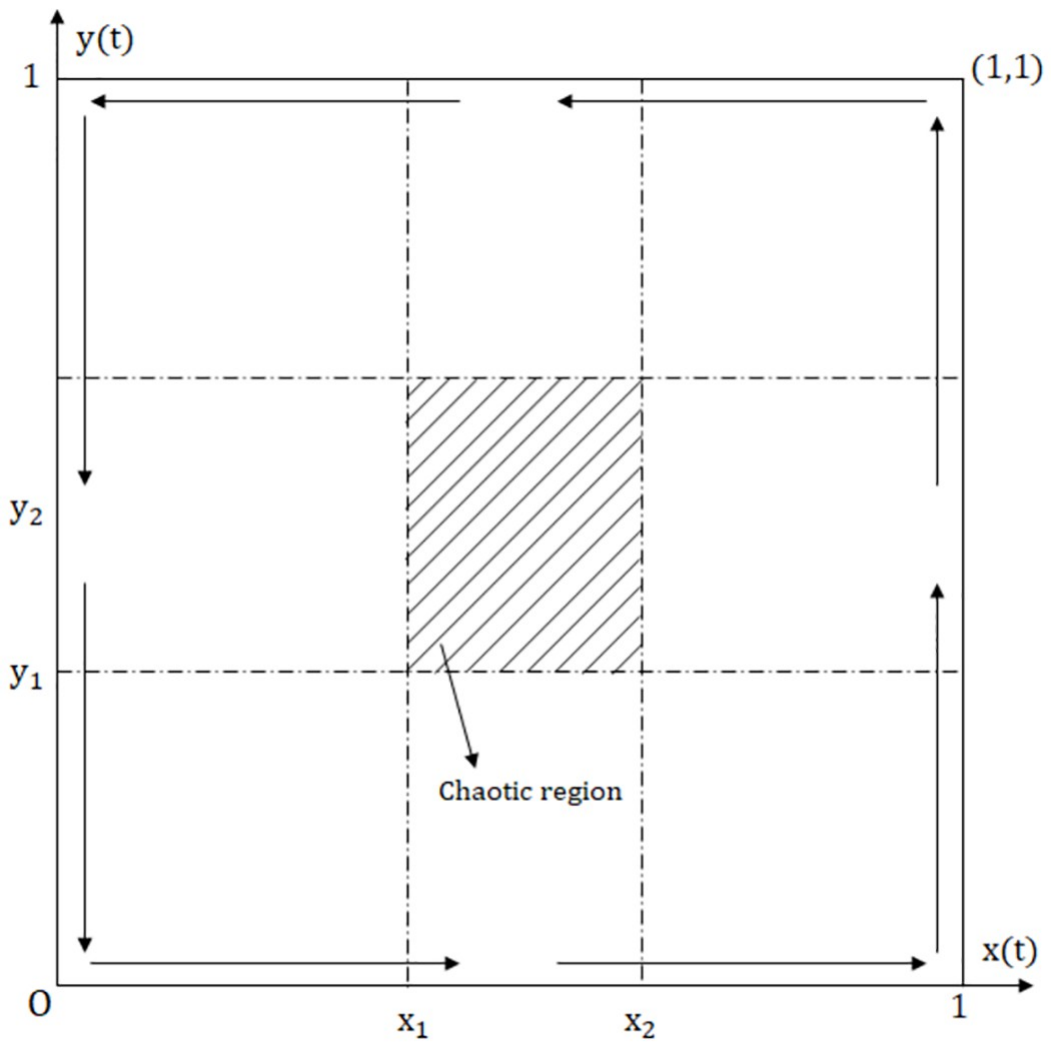
Chaotic region of binary stochastic game system.

**Proof:** According to Theorem 1, the range of values for zero and non-zero solutions concerning *x*(*t*) is: $\frac{qr+\left[S-c_{0}-\frac{b\left(1-ep\right)}{2\alpha }\right]+1}{qr}\leq y\left(t\right)\leq 1$ and $0\leq y\left(t\right)\leq \frac{qr+\left[S-c_{0}-\frac{b\left(1-ep\right)}{2\alpha }\right]-1}{qr}$. the range of values for zero and non-zero solutions concerning *y*(*t*) is: $0\leq x(t)\leq \frac{(2\beta \Delta h-d)-2\beta }{2\beta \varphi g}$ and $\frac{(2\beta \Delta h-d)+2\beta }{2\beta \varphi g}\leq x(t)\leq 1$. Therefore, the stability domain boundaries for *x*(*t*) and *y*(*t*) do not overlap entirely. There exists an uncovered chaos region as shown in Fig 2. The chaotic region represents the mutual influence of strategies between government departments and Internet platforms, exhibiting an intertwined oscillating state. The system can evolve toward various equilibrium points when *x*(*t*) and *y*(*t*) fall into this region.

### 4.2 Continuous strategy set evolutionary game

The evolutionary game model is based on the premise that the players are members of a population, and individuals within the population dynamically adjust their strategic choices through learning and imitation to improve their expected benefits. Therefore, the strategic choice probabilities within a population are continuous variables over a range rather than purely binary and discrete. The stability of game system evolution under continuous strategy sets can be analyzed by defining probability density functions for strategic choice by game agents [54]. Bomze and Potscher [55] introduced the concept of continuous strategies in the context of "generalized" mixed-strategy games. Van and Spreij [56] researched the correlation between evolutionary stability, the existence of a uniform invasion barrier, local superiority, and asymptotic stability in continuous strategy games. Ruijgrok and Ruijgrok [57] derived the partial differential equations for symmetric games with one-dimensional continuous strategy sets and analyzed the game stability solutions under the effect of mutation factors. The government department’s regulatory intensity *m* and the internet platform’s fair competition level *n* should be continuous variables in the range [0,1]. For the convenience of calculation, it is assumed that *m* and *n* obey the uniform distribution on [0,1]. In addition, the probability density functions for strategic choice are represented by *f*(*m*) and *f*(*n*), reflecting the game agents’ gradual nature of strategic choices.

Based on the model assumptions, the expected benefits for the government’s choice of regulatory intensity *m* and the Internet platform’s choice of fair competition level *n* can be expressed as follows:

$$EX\left(m\right)=\int_{0}^{1}\left[mS-{mc}_{0}-\frac{b\left(1-ep\right)}{2\alpha }m^{2}+nw-\left(1-n\right)v-(1-m)(1-n)qr\right]f\left(n\right)dn,$$ (14)

$$EY\left(n\right)=\int_{0}^{1}\left[H+\left(1-n\right)\Delta h-\frac{d}{2\beta }{\left(1-n\right)}^{2}-m\left(1-n\right)\varphi g\right]f\left(m\right)dm.$$ (15)

**Proposition 4:** Under the continuous strategy set, the direction to guide the strategic choices of the game agents toward the positive state (1,1) can be represented as follows:

xm1,t=yn1,t=1(m1=n1=1),xm2,t=yn2,t=0m2∈0,1,n2∈0,1. (16)

The threshold conditions are:

$$p>\frac{b-2\alpha \left[2\left(S-c_{0}\right)+qr\right]}{4eb},g>\frac{2\Delta h}{\varphi }.$$ (17)

**Proof:** In the game process, the government departments continuously adjust strategic choices to maximize payoff. The condition for guiding the government departments to increase regulatory intensity is that $\frac{\partial EX\left(m\right)}{\partial m}>0$ always holds. The first and second-order partial derivatives of Eq (14) for *m* can be calculated as follows.


$$\frac{\partial EX\left(m\right)}{\partial m}=\int_{0}^{1}\left[S-c_{0}-\frac{b\left(1-ep\right)}{\alpha }m+(1-n)qr\right]f\left(n\right)dn,$$ (18)

$$\frac{\partial^{2}EX\left(m\right)}{\partial m^{2}}=-\int_{0}^{1}\frac{b\left(1-ep\right)}{\alpha }f\left(n\right)dn.$$ (19)

It can be observed that, within the specified range of parameter values, $\frac{\partial^{2}EX\left(m\right)}{\partial m^{2}}<0$, indicating that $\frac{\partial EX\left(m\right)}{\partial m}$ is a decreasing function for *m* within the range *mϵ*[0,1], and it reaches its minimum value at *m* = 1.


$${\left.{\frac{\partial EX\left(m\right)}{\partial m}}_{min}=\int_{0}^{1}\left[S-c_{0}-\frac{b\left(1-ep\right)}{\alpha }m+(1-n)qr\right]f\left(n\right)dn\right|}_{m=1}.$$ (20)

When ${\frac{\partial EX\left(m\right)}{\partial m}}_{min}>0$, it means that *EX*(*m*) increases with the increase of *m* value, and the system eventually evolves toward active regulation. Since *m* follows a uniform distribution in the interval [0,1], then $\int_{0}^{1}mf\left(m\right)dm=E\left(m\right)=\frac{1}{2}$. Therefore, the condition for the government department’s strategic choice toward active regulation is $S-c_{0}-\frac{b\left(1-ep\right)}{\alpha }+\frac{qr}{2}>0$. Similarly, the condition for the Internet platform’s strategic choice toward fair competition is $-\Delta h+\frac{\varphi g}{2}>0$.


$$S-c_{0}-\frac{b\left(1-ep\right)}{\alpha }+\frac{qr}{2}>0,-\Delta h+\frac{\varphi g}{2}>0.$$ (21)

Eq (21) can be transformed to obtain Eq (17), and Proposition 4 is proved.

In the same way, the direction to guide the strategic choices of the game participants toward the positive state (0,0) can be represented as follows:

$$S-c_{0}+\frac{qr}{2}<0,-\Delta h+\frac{d}{\beta }+\frac{\varphi g}{2}<0.$$ (22)

**Proposition 5:** Under the continuous strategy set, the evolutionary trend of the game system toward the positive state (1,1) is hindered, and the evolutionary pathway is altered. However, increasing public participation can help mitigate or even eliminate the hindrance.

**Fig 3:**
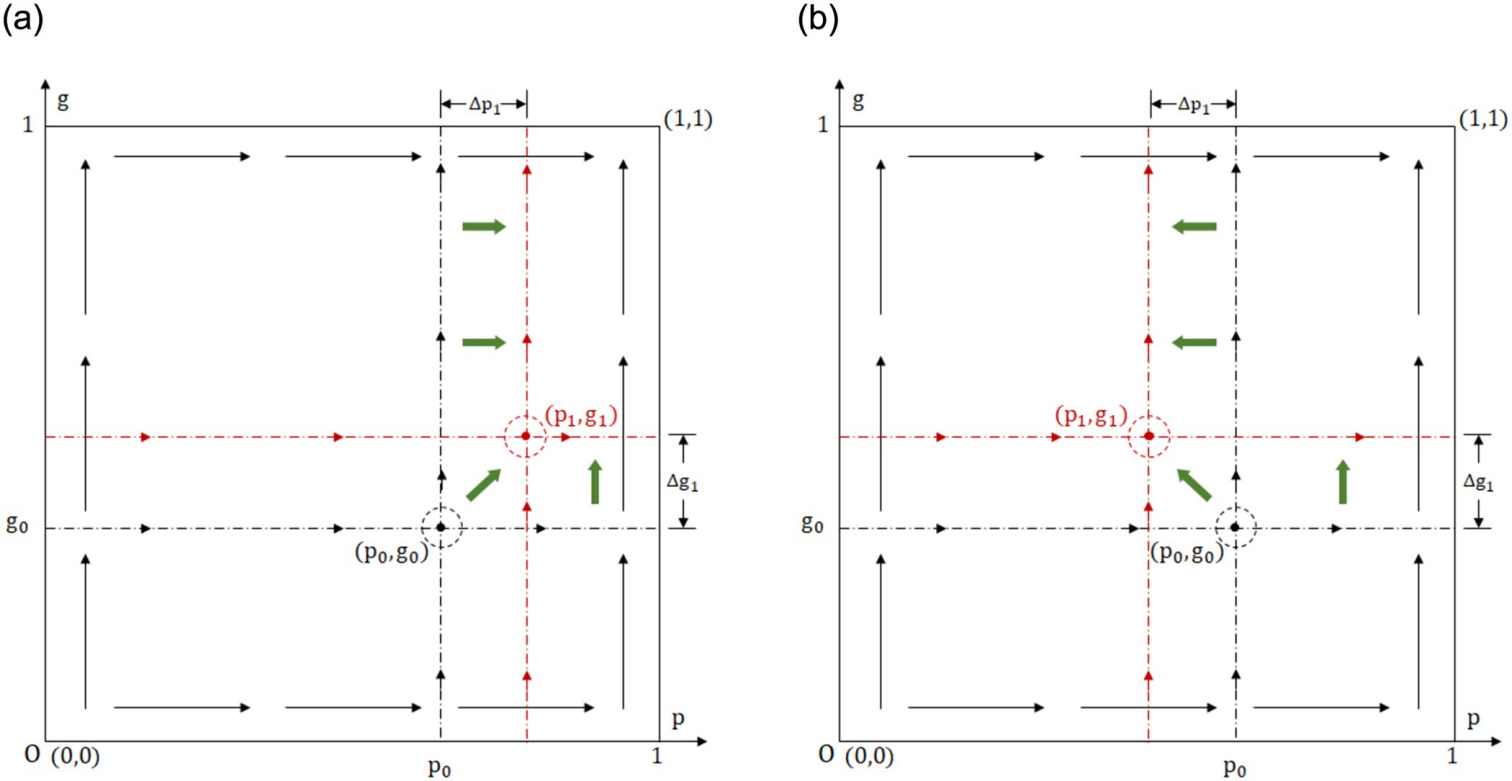
The evolutionary pathway of binary deterministic and continuous strategy set game system. (a) The case of Δ*p*_1_ > 0(*q* < *q*_1_). (b) The case of Δ*p*_1_ < 0(*q* > *q*_1_).

**Proof:** Let $p_{1}=\frac{b-2\alpha \left[2\left(S-c_{0}\right)+qr\right]}{4eb},g_{1}=\frac{2\Delta h}{\varphi },p_{0}=\frac{b-2\alpha (S-c_{0})}{be},g_{0}=\frac{2\beta \Delta h-d}{2\beta \varphi }.\Delta g=g_{1}-g_{0}=\frac{2\beta \Delta h+d}{2\beta \varphi }>0$ means the threshold of *g* increases, and the condition range is tightened. $\Delta p_{1}=p_{1}-p_{0}=\frac{\alpha \left(2S-{2c}_{0}-qr\right)}{2eb}$, the positive and negative sign cannot be determined. Setting Δ*p*_1_ = 0, the public participation threshold $q_{1}=\frac{2\left(S-c_{0}\right)}{r}$ can be obtained. When *q* > *q*_1_, Δ*p*_1_ < 0 means the *p*_1_ axis shifts to the left, lowering the threshold and expanding the condition range. When *q* < *q*_1_, Δ*p*_1_ > 0, the *p*_1_ axis shifts to the right, raising the threshold and tightening the condition range. An increase in parameter thresholds means the game system’s evolution toward the positive state is hindered. Conversely, decreasing parameter thresholds representing the game system’s evolution toward the positive state becomes easier. Since *p*,*gϵ*[0,1], a two-dimensional coordinate system can be constructed with the horizontal axis as *p* and the vertical axis as *g*. The evolutionary pathways of the binary deterministic and stochastic game systems can be compared in the two-dimensional coordinate system. as shown in Fig 3(a) and 3(b).

**Fig 4:**
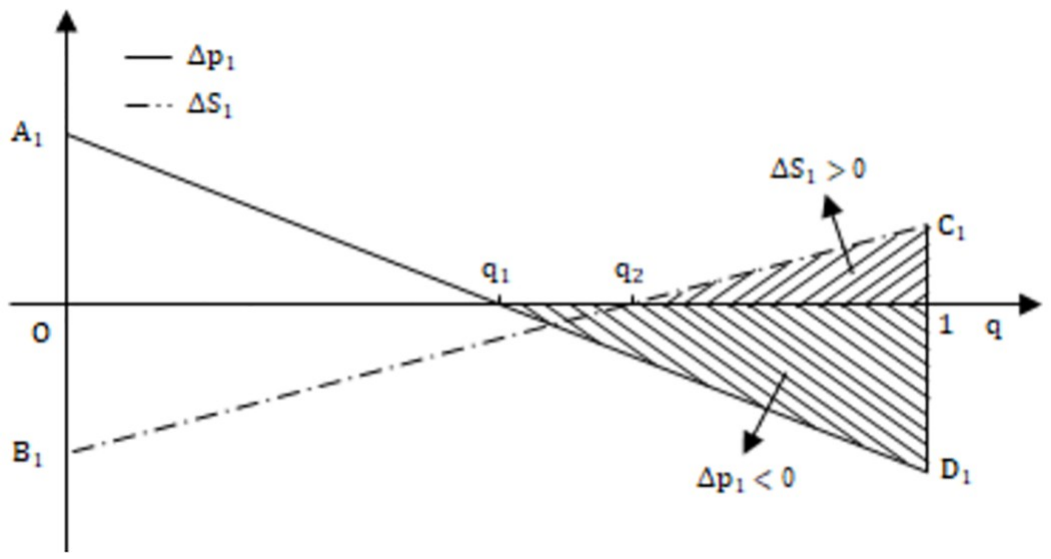
The promotion effect of public participation *q* on the positive state.

Public participation guides government departments to strengthen regulation through reputation mechanism, and has a certain substitution effect with industry associations. When public participation is low, the reputation loss suffered by the government is slight, and the reputation mechanisms play a limited role, resulting in limited positive effects on the system’s evolution. As public participation increases, the *p*_1_ axis shifts to the left, and the probability of the system evolving toward the positive state gradually increases. Now, consider an extreme scenario: Is it possible to find a sufficiently large *g* value that makes the *p*_1_ axis shift to the left and counteract the negative effect caused by the upward shift of the *g*_1_ axis? It can be seen from Fig 3 that the area enclosed by (*p*_0_,*g*_0_), (*p*_1_,*g*_1_), and the coordinates point (1,1) reflects the expected probability of binary deterministic and continuous strategy set game systems evolving toward the positive state. The area difference Δ*S*_1_ = − Δ*p*_1_ (1 − *g*_1_) − Δ*g*_1_ (1 − *p*_0_), setting Δ*S*_1_ = 0, we can obtain the public participation threshold *q*_2_. From Fig 4,when parameter *q* is greater than *q*_1_, Δ*p*_1_ changes from positive to negative, indicating a decrease in the parameter threshold. When parameter *q* is greater than *q*_2_, Δ*S*_1_ changes from negative to positive, implying that the positive effect of public participation outweighs the negative effect of continuous strategy set. In Fig 4, $A_{1}=\frac{2\alpha }{be};B_{1}=-\frac{2\alpha }{be}\left(1-g_{1}\right)-\Delta g_{1}\left(1-p_{0}\right);C_{1}=\frac{2\alpha \left(r-1\right)}{be}\left(1-g_{1}\right)-\Delta g_{1}\left(1-p_{0}\right);D_{1}=\frac{-2\alpha \left(r-1\right)}{be};q_{1}=\frac{2\left(S-c_{0}\right)}{r};q_{2}=\frac{2\left(S-c_{0}\right)}{r}+\frac{2eb\Delta g_{1}\left(1-p_{0}\right)}{r\alpha \left(1-g_{1}\right)}$.

In the continuous strategy set game system, public participation lowers the threshold of parameter *p* and increases the probability of the system converging to the positive state. According to Eqs (9) and (12), and (17), public participation is a critical factor in locking the continuous strategy set game system’s ESS into the positive state. However, it does not apply to the binary strategy set game system. Even though public participation is not explicitly reflected in the threshold conditions of the binary strategy set game system, it still significantly affects the evolutionary pathway and direction of the system. Based on the replication dynamic Eqs (5) and (10) and Theorem 1, when *x*(*t*) stabilizes at 1, public participation cannot exert an influence through the reputation mechanism. However, when *x*(*t*) deviates from 1, public participation will hinder the binary strategy set game system from evolving toward the negative state and promote the system to evolve toward the positive state.

From a real-world perspective, the public is the primary group suffering from IPUC. When the public has a strong safeguard awareness of their rights and a low tolerance for IPUC, they are likelier to report and complain, hoping that government authorities will take effective administrative measures. If the government does not take action in such cases, it can seriously damage social credibility and authority and even trigger vicious negative online public sentiment. Therefore, government departments adopt stricter regulatory measures in typical unfair competition events with significant negative social effects and harm to public interests. For example, in April and October 2021, the Chinese government imposed fines of 4% of their domestic sales revenue on Alibaba and 3% on Meituan for their forced exclusivity monopoly practices, totaling 18.228 billion RMB and 3.442 billion RMB.

**Proposition 6:** The game agents’ strategic choices are not a pure binary set of 0 or 1 under the continuous strategy set. The optimal solution (*m*_0_, *n*_0_) can be found on the (0,1) interval, maximizing the expected benefits of game agents. In this case, (*m*_0_, *n*_0_) will become the stable strategy.

**Proof:** Compared with Eqs (21) and (22), it is found that the boundary of the parameter conditions does not overlap. When the parameter values satisfy Eq (23), *m* and *n* will stabilize near specific values and not evolve toward 1 or 0.


$$0<S-c_{0}+\frac{qr}{2}<\frac{b\left(1-ep\right)}{\alpha },\Delta h-\frac{d}{\beta }<\frac{\varphi g}{2}<\Delta h.$$ (23)

According to Eq (14), the expected benefits of the government department choosing the regulatory intensity *m* is:

$$-\frac{b\left(1-ep\right)}{2\alpha }m^{2}+\left(S-c_{0}+\frac{qr}{2}\right)m+\frac{w-v-qr}{2}.$$ (24)

**Fig 5:**
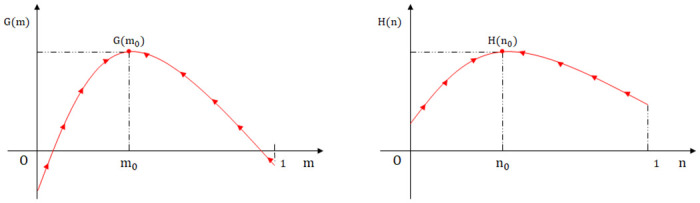
The graphs of the quadratic concave functions *G*(*m*) and *H*(*n*).

Eq (24) is a downward-opening quadratic function with a maximum value. Let *G*(*m*) = *am*^2^ + *bm* + *c*. According to the properties of quadratic functions, the coordinates of the maximum value for *G*(*m*) can be found at $m_{0}=-\frac{b}{2a}$. Substituting the values: $m_{0}=\frac{\alpha \left(2S-2c_{0}+qr\right)}{2b\left(1-ep\right)}$. Similarly, the maximum value coordinate of the Internet platform benefits function *H*(*n*) can be obtained as $n_{0}=1-\frac{2\beta \Delta h-\beta \varphi g}{2d}$. According to the parameter value condition of Eq (23), *m*_0_, *n*_0_ ∈(0,1) can be obtained. Therefore, there exists an optimal solution (*m*_0_, *n*_0_) maximizes the expected benefits, and Proposition 6 is proved. By substituting 0 and 1 into the functions *G*(*m*) and *H*(*n*) to obtain the function endpoint values. Combined with the conditional range of parameter values, sketch the function graphs roughly shown in Fig 5.

The expected benefits increase initially and then decrease with the increase of parameters *m* and *n*, exhibiting the characteristics of a concave function. According to Proposition 6, compared with the binary strategy set, the continuous strategy set evolutionary game model better aligns with the regulatory practices of IPUC. On the one hand, government departments exhibit different regulatory intensity across different fields, showing the characteristics of "couple hardness with softness." They apply more robust regulatory measures and impose heavier penalties on mature platform economy models such as e-commerce and food delivery. Simultaneously, out of consideration of supporting emerging industries and protecting innovation, the regulation intensity of emerging platform economy models such as community group buying is weak, and the penalties are minor. For instance, in March 2021, the Chinese government fined community group buying platforms like Meituan and Pinduoduo only a few million yuan for their unfair price competition while imposing fines of tens of billions of yuan on Meituan and Alibaba for "exclusivity monopoly" practices. On the other hand, the regulatory intensity of government departments varies at different times. After significant incidents of unfair competition with substantial negative impacts and major public reactions, the regulatory intensity remains high for a certain period and gradually tapers off later. From the perspective of Internet platforms, cross-border competition is a common phenomenon in the platform economy, and the business scope covers multiple fields. In business fields where knowledge and technical capabilities are dominant, market profit margins are high, regulatory intensity is low, and unfair competition is relatively high. In contrast, in other business fields, it is relatively low.

**Proposition 7:** Although the parameters have different magnitudes of effect on the game system, their effect direction is consistent in the three evolutionary game scenarios described above. As long as the parameter values are maintained above or below certain thresholds, the result of the system evolving toward the positive state remains unchanged.

**Proof:** By analyzing the correlation between *p*, *g* and parameters in each scenario, the effect of parameters on the evolution of the game system toward the positive state can be determined. The positive correlation between *p*, *g* and parameters represents that the threshold is raised, and the range of conditions becomes more restrictive, hindering the game system’s evolution toward the positive state. Conversely, the negative correlation represents that the threshold is lowered, and the range of conditions expands, making it easier for the game system to evolve toward the positive state. Taking *α*, *e* and *β* as examples, $\frac{\partial p}{\partial \alpha }<0,\frac{\partial p}{\partial e}<0,\frac{\partial g}{\partial \beta }>0$, indicating that *α* and *e* have a positive effect on the game system’s evolution, while *β* has a negative effect. Following this method, we can determine each parameter’s positive or negative effects on the game system’s evolution, as shown in Table 4.

**Table 4:** The effect of parameters on the evolution of the game system toward the positive state.

Parameters	*S*	*c*_0_	*α*	*b*	*e*	Δ*h*	*β*	*d*	*q*	*r*
Effect direction	↑	↓	↑	↓	↑	↓	↓	↑	↑	↑

(1) Regulatory ability *α*. The regulatory decisions of government departments are affected by their regulatory capabilities and costs. The government departments’ investigation and evidence-collection processes are lengthy and challenging due to the diverse and covert nature of IPC’s methods and approaches, significantly increasing regulatory costs. Government departments should keep pace with the times, establish comprehensive regulatory databases, and leverage emerging technologies such as big data and artificial intelligence to innovate regulatory methods, effectively enhancing regulatory capabilities.

(2) Power Space *e*: For government departments, it is essential to strike the right balance in granting NGO power space. If the power space is too large, it can easily lead to corruption, exchange of interests, and abuse of authority. However, if the power space is too small, it may hinder NGOs from effectively contributing to the co-governance system. Therefore, the government needs to position the role of "collaborator" of NGOs. For regulatory matters that are non-core, highly professional, and consistent with the capabilities and advantages of NGOs, the government can appropriately decentralize and hand them over to NGOs to handle on their behalf.

(3) Unfair competitive ability *β*. The larger *β* indicates that the Internet platform has a larger scale, higher market share, and more significant advantages in terms of users, technology, and knowledge. Such platforms are more inclined to use unfair competition methods to gain excessive profits. In common IPUC cases, the involved agents are usually top-tier Internet platforms. Therefore, government departments can select top-tier Internet platforms for focused monitoring and increase penalties for unfair competition on such Internet platforms to achieve the purpose of "killing chickens to scare monkeys."

## 5 Case and simulation analysis

This section utilizes case studies and MATLAB to simulate and analyze the evolutionary paths of the game system under random disturbances and continuous strategy sets, as well as the impact of critical parameters on the strategic choices of game participants.

TCOM is a well-known Internet travel platform in China, providing users with travel services such as online hotel reservations, flight ticket purchases, and travel route planning. As of December 2023, TCOM has 106 million active users. The market size of Internet travel is substantial, with competitors such as Qunar, Alibaba Fliggy, and Weekend Hotel being TCOM’s main competitors. In recent years, TCOM has implemented unfair competition practices such as price discrimination and forced exclusivity monopoly to increase operating profits and frustrate the competitor. (1) In July 2020, a platform user exposed TCOM’s price discrimination behavior to the media. The actual hotel price was 1400 yuan, but the TCOM price and user payment were 3000 yuan. The platform user appealed to the court against TCOM’s illegal business practices to safeguard rights and interests. Ultimately, the court ruled that TCOM had to compensate the platform user for three times the price difference. (2) In October 2021, the weekend hotel accused TCOM of abusing its market dominance to implement the forced exclusivity monopolistic practices and complained to the 12315 platform. This incident spread rapidly on the Chinese Internet, tarnishing TCOM’s market reputation and leading to the loss of some TCOM users. TCOM’s unfair competition practices violated the law of consumer protection and anti-unfair competition. In response, government departments (Market Supervision Bureau, Industry and Commerce Bureau) collaborated with industry associations (Internet Association, Consumer Association, Payment Association) to investigate TCOM and imposed fines totaling over a million yuan.

This paper refers to standard methods in the evolutionary game theory field to assign parameter values due to the difficulty in collecting empirical data corresponding to the theoretical model parameters [58, 59]. We follow two principles when setting the initial parameter values. (1) It conforms to the logical relationship between parameters. (2) Consistency with the actual situation of IPUC regulation. The initialization value of model parameters are shown in Table 5.

**Table 5:** The initialization value of model parameters.

Parameters	*S*	*c*_0_	*b*	*e*	*r*	Δ*h*	*d*	*φ*
Value	5	2	4	0.8	20	6	4	40

### 5.1 Binary strategy set evolutionary game

**Fig 6:**
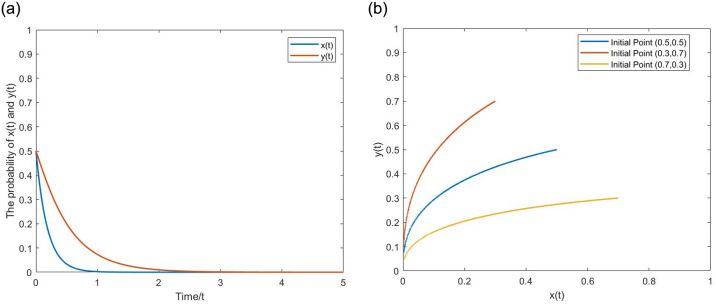
The evolutionary pathway (0,0) of binary deterministic game system. (a) One initial point. (b) Multiple initial points.

**Fig 7:**
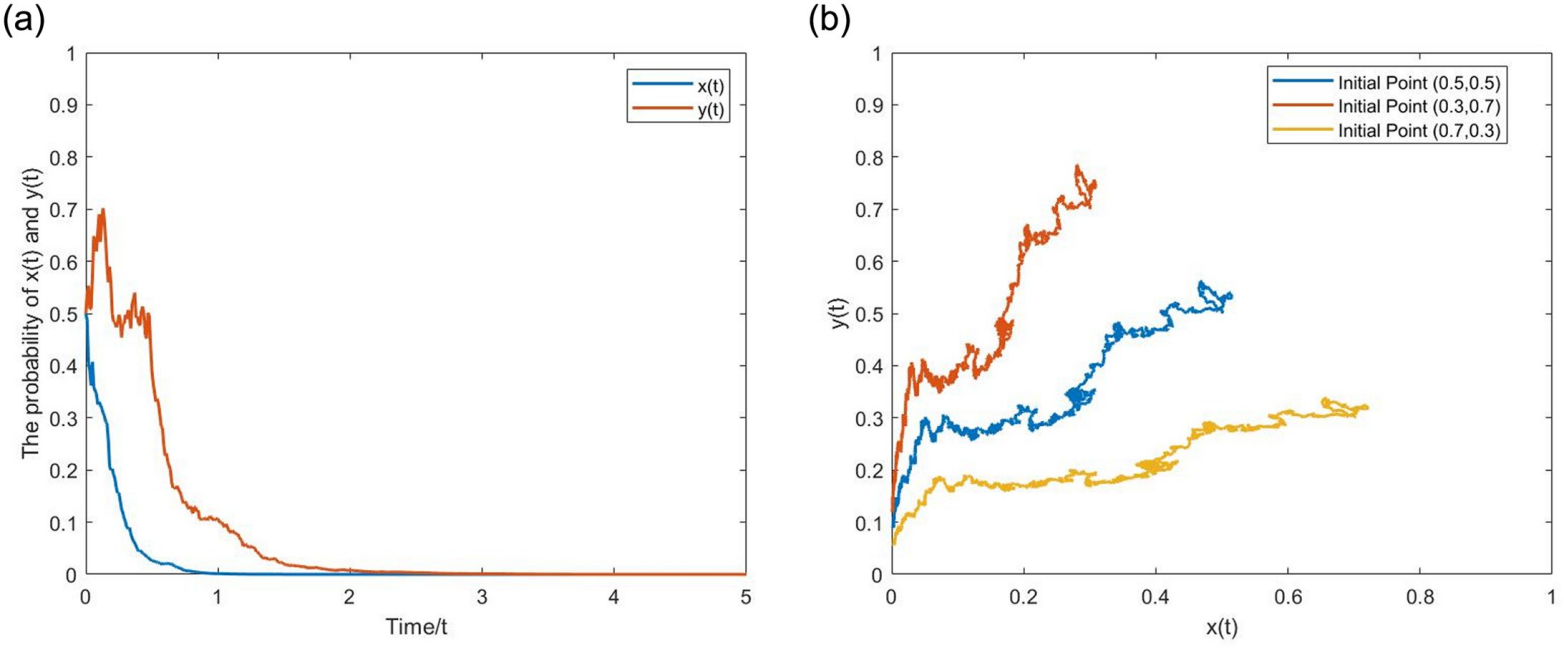
The evolutionary pathway (0,0) of binary stochastic game system. (a) One initial point. (b) Multiple initial points.

**(1) Evolutionary pathway (0,0).** Let *α* = 0.2, *p* = 0.2, *q* = 0.01, *β* = 0.5, *g* = 0.025. *σ* = 0,0.5 correspond to the deterministic and stochastic game systems, respectively. From the Figs 6 and 7, when the NGO participation and penalty intensity are relatively low, as time evolves, the probabilities of *x*(*t*) and *y*(*t*) show a downward trend, eventually approaching 0. The strategy set is (passive regulation, unfair competition). Upon comparison, it is evident that the evolutionary trajectory of the stochastic game system is not smooth and exhibits fluctuations with ups and downs, reflecting the interference of external stochastic factors. Meanwhile, *x*(*t*) and *y*(*t*) remain stable after reaching (0,0), demonstrating the characteristic of initial value problem zero solutions having k-order moment exponent stability.

**Fig 8:**
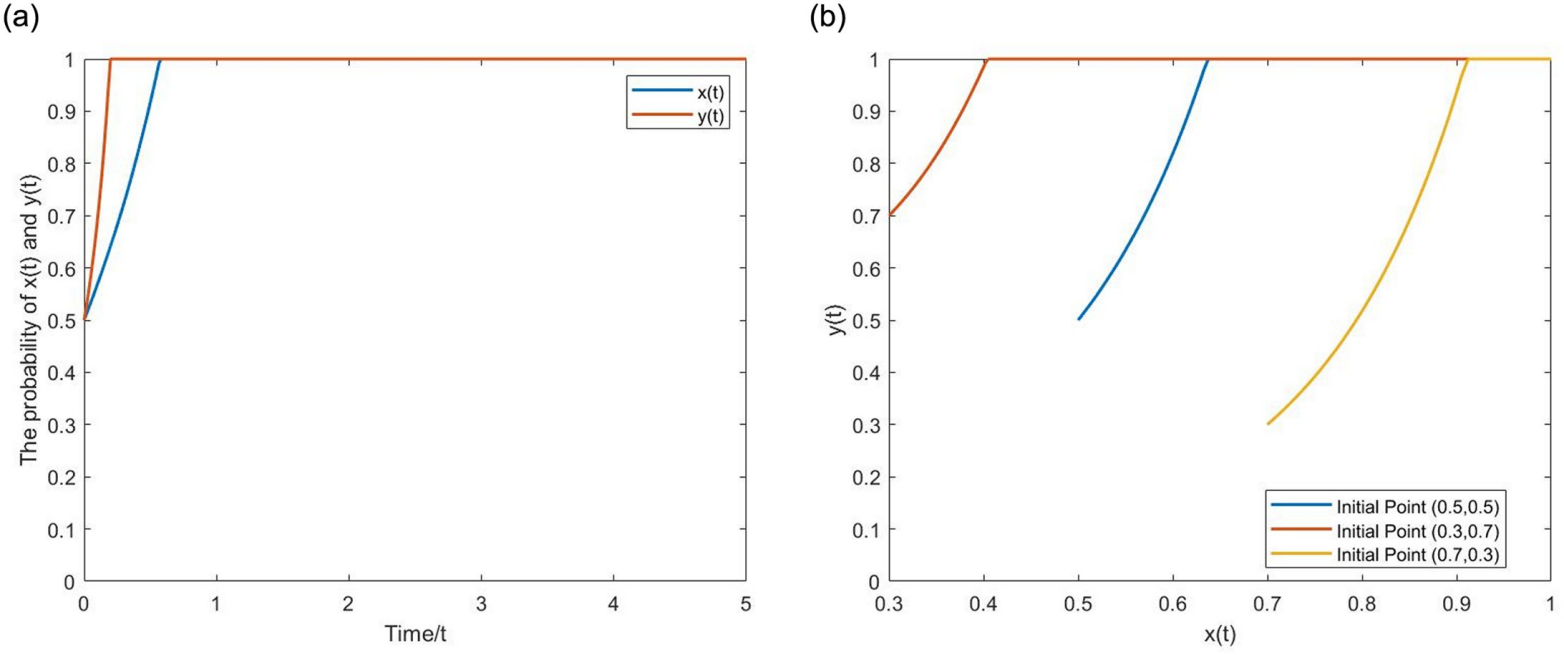
The evolutionary pathway (1,1) of binary deterministic game system. (a) one initial point. (b) multiple initial points.

**Fig 9:**
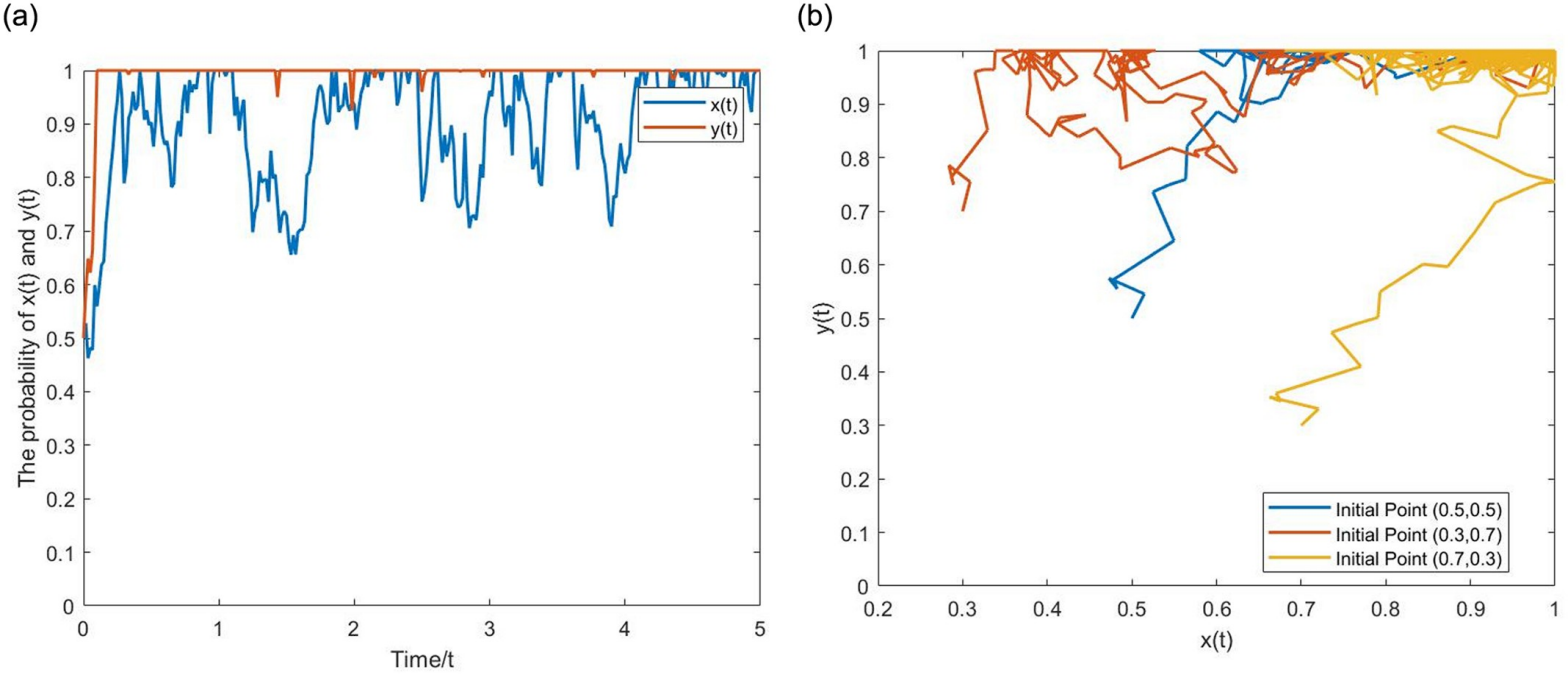
The evolutionary pathway (1,1) of binary stochastic game system. (a) one initial point. (b) multiple initial points.

**Fig 10:**
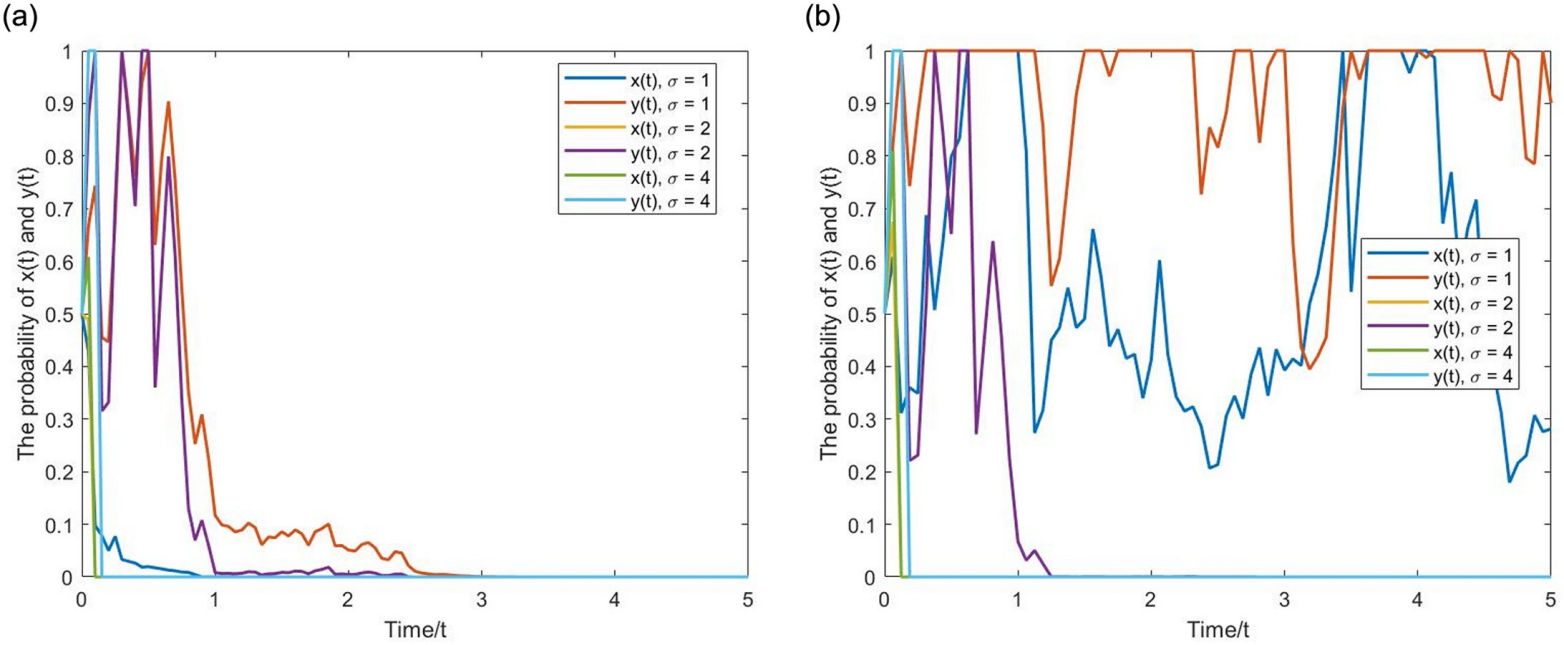
The inhibitory effect of stochastic disturbance intensity *σ* on the the positive state. (a) Equilibrium point (0,0). (b) Equilibrium point (1,1).

**(2) Evolutionary pathway (1,1).** Let *α* = 0.4, *p* = 0.8, *q* = 0.01, *β* = 0.5, *g* = 0.25. *σ* = 0,0.5 correspond to the deterministic and stochastic game systems, respectively. From the Figs 8 and 9, when the NGO participation and penalty intensity are relatively high, as time evolves, the probabilities of *x*(*t*) and *y*(*t*) show an upward trend, eventually approaching 1. The strategy set is (active regulation, fair competition). Upon comparison, it is evident that *x*(*t*) and *y*(*t*) of the stochastic game system still exhibit inevitable fluctuations after reaching (1,1). The positive state (1,1) under stochastic disturbance exists within a certain noise intensity. When the noise intensity increases to a certain level, it can still significantly affect the stability of the game system, even breaking the stable state and leading it to evolve toward (0,0), demonstrating the characteristic of initial value problem’s Non-zero solutions having k-order moment exponent stability. The following Fig 10 provides a more visual representation of the influence of noise.

As indicated above, the evolutionary curve of the stochastic game system has more twists and turns, and the pathway of evolution becomes longer. This suggests that considering stochastic disturbances, in multiple repetitions of the game process, the strategic choices of boundedly rational game agents are wavering, and they often make decisions that deviate from the direction of ESS. However, as long as the parameters meet the range of ESS value conditions, the final evolutionary results will not change.

**(3) The effect of the stochastic disturbance intensity.** Let *σ* = 1,2,4, with parameter values consistent with (0,0) and (1,1) above. From the Fig 10(a) and 10(b), as the stochastic disturbance intensity increases, the time for game agents’ strategy to evolve toward (0,0) and (1,1) shortens, and the curve fluctuations increase. The stability of the game system at (1,1) becomes worse, and *x*(*t*) and *y*(*t*) deviates further from 1. When the stochastic disturbance increases to *σ* = 2,4, the game system eventually evolves toward (0,0). Therefore, on the one hand, stochastic disturbances shorten the evolutionary time and promote (hinder) the evolution of the game system to the negative (positive) state. On the other hand, when the stochastic disturbance reaches a certain level, (1,1) will no longer remain stable, and the game system’s ESS changes to the negative state.

**Fig 11:**
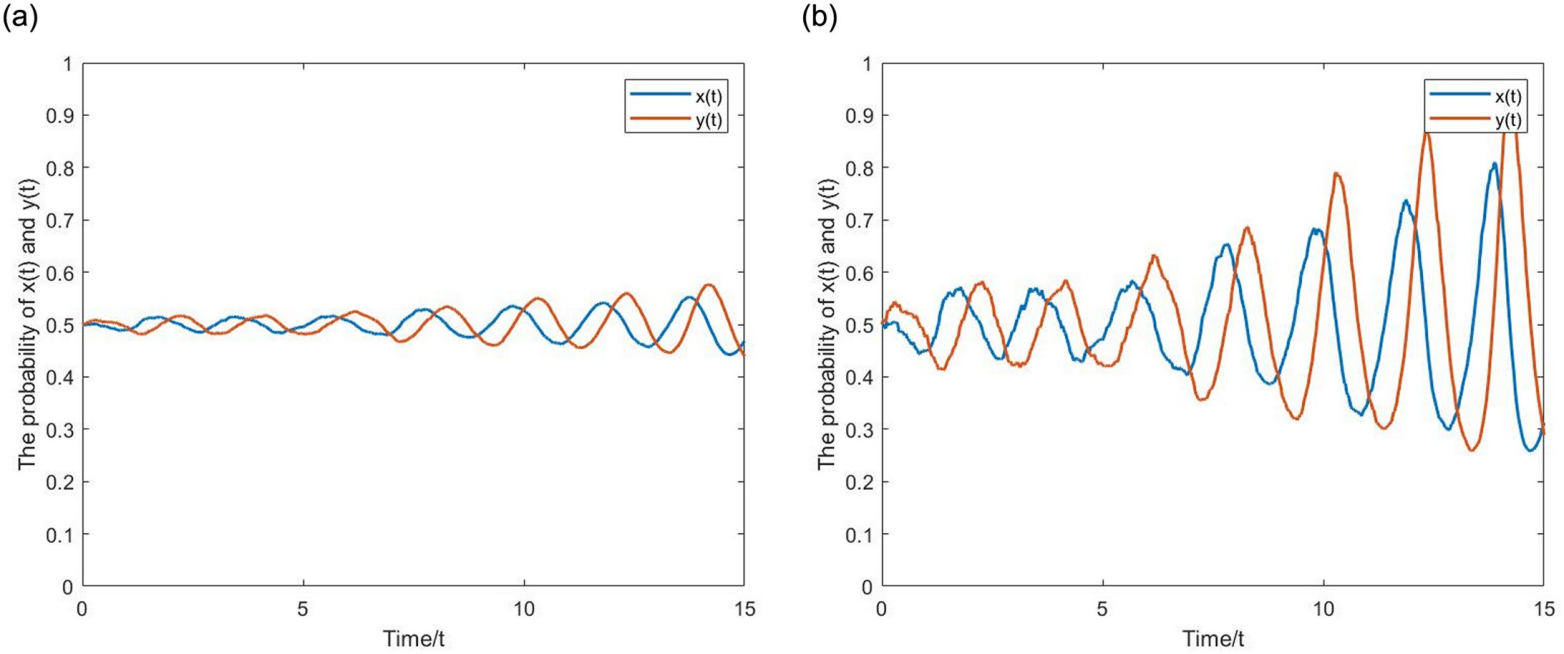
The Evolutionary pathways of oscillatory motion in the chaotic state. (a) *σ* = 0.01. (b) *σ* = 0.05.

**(4) Chaotic State:** Let *α* = 0.2, *p* = 0.55, *q* = 0.25, *β* = 1, *g* = 0.2, *σ* = 0.01,0.05. According to Proposition 3, take *x*(*t*) = *y*(*t*) = 0.5, satisfying 0 ≤ *x*_1_ ≤ *x*(*t*) ≤ *x*_2_ ≤ 1,0 ≤ *y*_1_ ≤ *y*(*t*) ≤ *y*_2_ ≤ 1. From the Fig 11(a) and 11(b), the evolutionary curve of the game system oscillates up and down around the initial point, showing a tendency for strategic choice to evolve toward multiple equilibrium points, and the amplitude of the curve increases with time. At the same time node, with a larger stochastic disturbance intensity, the amplitude becomes larger.

**Fig 12:**
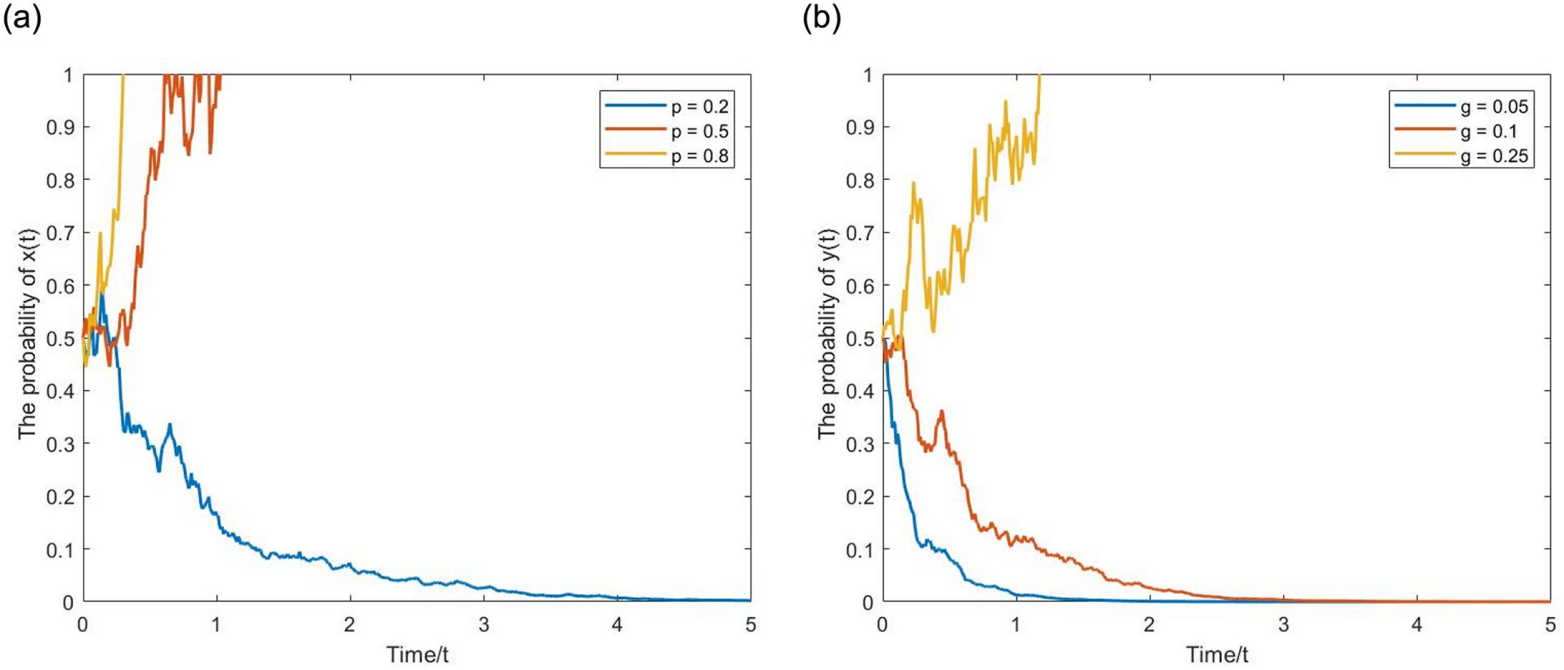
The positive effect of parameter *p* on *x*(*t*) and parameter *g* on *y*(*t*). (a) *x*(*t*). (b) *y*(*t*).

**(5) The effect of NGO participation *p* on *x*(*t*).** The initial value *x*(*t*) = 0.5. Let *α* = 0.25, *q* = 0.25, *σ* = 0.5, *y*(*t*) = 0.5, and the evolutionary simulation diagram is shown in Fig 12(a) and 12(b). When the NGO participation is relatively low, for example, *p* = 0.2, the expected benefits of choosing active regulation by the government department, given the limited cost-sharing by NGOs, are still lower than choosing passive regulation. Therefore, the evolutionary curve gradually tends toward 0. However, when the NGO participation increases to *p* = 0.5, 0.8, the regulatory cost decreases significantly, leading the government department to choose active regulation, and the evolutionary curve gradually tends toward 1.

**(6) The effect of penalty intensity *g* on *y*(*t*).** The initial value *y*(*t*) = 0.5. Let *β* = 1, *σ* = 0.5, *x*(*t*) = 0.5, and the evolutionary simulation diagram is shown in Fig 12. When the penalty intensity is relatively low, *g* = 0.05, the deterrent and constraint effect on the internet platform is limited. The expected benefits of choosing unfair competition strategy are still higher than choosing fair competition. Therefore, the evolutionary curve gradually tends toward 0. However, when the penalty intensity increases to *g* = 0.1, 0.25, the unfair competition strategy will face severe penalties, leading the internet platform to choose fair competition strategy, and the evolutionary curve gradually tends toward 1.

### 5.2 Continuous strategy set evolutionary game

#### (1) The effect of *m* on *EX*(*m*) under different *p* values

**Fig 13:**
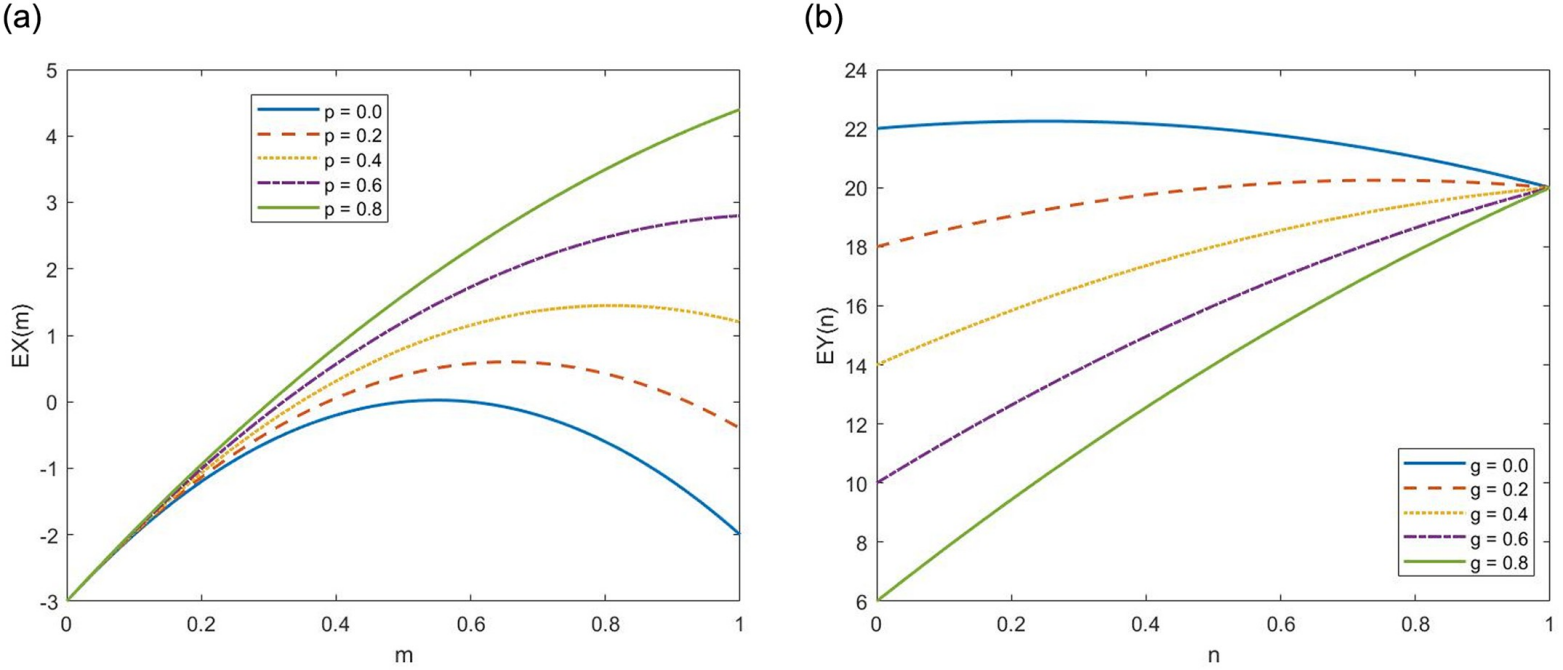
The inversed U-shaped and monotonic correlation between expected returns *EX*(*m*), *EY*(*n*) and parameters *m*, *n*. (a) *EX*(*m*). (b) *EY*(*n*).

Let *α* = 0.2, *q* = 0.8, *w* = 15, *v* = 5. When the *p* value is low, for instance, *p* = 0,0.2,0.4, the expected benefits *EX*(*m*) exhibit an initial increase followed by a decreasing trend with the regulatory intensity *m* increases. From the Fig 13(a), after multiple repetitions of the game, the regulatory intensity m stabilizes at the point *m*_0_(*m*_0_ < 1) corresponding to the peak of the curve and does not reach the point *m* = 1. As the *p* value increases to *p* = 0.6,0.8, the expected benefits *EX*(*m*) increase with the regulatory intensity *m*. After multiple repetitions of the game, the regulatory intensity m stabilizes at the point *m* = 1.

#### (2) The effect of *n* on *EY*(*n*) under different *g* values

Let *H* = 20, *β* = 0.5. When the *g* value is low, for instance, *g* = 0,0.2, the expected benefits *EY*(*n*) exhibit an initial increase followed by a decreasing trend with the fair competition level *n* increases. From the Fig 13(b), after multiple repetitions of the game, the fair competition level *n* stabilizes at the point *n*_0_(*n*_0_ < 1) corresponding to the peak of the curve and does not reach the point *n* = 1. As the *g* value increases to *g* = 0.4,0.6,0.8, the expected benefits *EY*(*n*) increase with the fair competition level *n*. After multiple repetitions of the game, the fair competition level *n* stabilizes at the point *n* = 1.

As indicated above, the critical parameters *p* and *g* affect the strategic choices of game agents by modifying their benefit functions. Since the benefit functions are quadratic concave functions, when *p* and *g* are at a low level, the symmetry axis of the benefit function is located in the (−∞, 1) interval, meaning that the optimal strategic choice for game agents is 0 < *m*_0_, *n*_0_ < 1. As *p* and *g* values increase, the symmetry axis of benefit functions shifts to the right until it is located in the [1, + ∞] interval. When this occurs, the optimal strategic choice for game agents is *m*_0_, *n*_0_ = 1, and the game system will evolve toward the positive state.

## 6 Conclusion and prospect

### 6.1 research conclusions

This paper explores the strategic interaction mechanism between government departments and Internet platforms in IPUC regulation when considering stochastic disturbances and continuous strategy sets. It utilizes model-solving and simulation analysis to investigate the evolutionary pathway of the game system toward the positive state and the critical parameter value conditions. The primary conclusions are as follows.

(1) the binary deterministic evolutionary game system has multiple equilibrium points, and the evolutionary trajectory is smooth. Maintaining the participation level of NGOs and penalty intensity above the threshold level, will make the equilibrium point (1,1) become the ESS.

(2) In the binary stochastic evolutionary game system, the evolutionary trajectory becomes tortuous and fluctuating. The equilibrium point (0,0) remains stable, but the equilibrium point (1,1) is unstable and requires more stringent parameter conditions. Furthermore, the equilibrium point (1,1) exists under a certain noise intensity. As the noise intensity increases, the equilibrium point (1,1) becomes increasingly unstable, while the equilibrium point (0,0) remains unaffected. This indicates that stochastic disturbances hinder the convergence of the game system to the positive state, and the IPUC "multi-agent co-governance" system exhibits specific vulnerability.

(3) In the continuous strategy set evolutionary game system, the evolutionary trend of the system towards the positive state is hindered, which is manifested in the increase of the threshold for penalty intensity. However, the threshold for participation level of NGOs decreases as public participation increases. In other words, increasing public participation is advantageous for mitigating or even eliminating obstacles. The strategy choices of government departments and Internet platforms are more flexible and resilient rather than a pure binary set of 0 or 1. As the participation level of NGOs and penalty intensity decrease below the threshold, strategy choices will gradually stabilize at (*m*_0_, *n*_0_).

(4) Public participation is a critical condition parameter for locking the continuous strategy set evolutionary game system into the positive state. However, it does not apply to binary strategy set evolutionary game systems. In other words, when the strategy space of the game system is expanded to a continuous set, the positive role played by public participation becomes more significant. Regardless of the evolutionary game scenario, the direction of all parameter’s influence on the game system is consistent.

### 6.2 Research prospects

This paper still has certain limitations and requires further research and optimization. On the one hand, introducing NGOs and the public into the game system through participation levels, it would be possible in future studies to directly construct three-party or four-party game models involving the agents mentioned above, thereby enhancing the precision of the analysis process. On the other hand, under the continuous strategy set evolutionary game, for the sake of calculation convenience, it is assumed that the strategic choices of government departments and Internet platforms follow a uniform distribution. However, a normal distribution would be more consistent with reality. Subsequent research could delve deeper into these aspects.

## Appendix

### Appendix 1 Solution of binary stochastic evolutionary game

*f*(*t*, *y*(*t*)) ≤ −*y*(*t*),Substitute *f*(*t*, *y*(*t*)) to get:$y\left(t\right)\left[\left(-\Delta h+\frac{d}{2\beta })+\varphi gx(t\right)\right]\leq -y(t)$. because 0 ≤ *y*(*t*) ≤ 1,the equation can be simplified to:$\left[\left(-\Delta h+\frac{d}{2\beta })+\varphi gx(t\right)\right]+1\leq 0$,namely:$\left[\left(-\Delta h+\frac{d}{2\beta }\right)\right]+1\leq -\varphi gx(t)$. Because 0 ≤ *x*(*t*) ≤ 1,namely:$\left[\left(-\Delta h+\frac{d}{2\beta }\right)\right]+1\leq -\varphi g$.

*f*(*t*, *y*(*t*)) ≥ *y*(*t*), Substitute *f*(*t*, *y*(*t*)) to get:$y\left(t\right)\left[\left(-\Delta h+\frac{d}{2\beta })+\varphi gx(t\right)\right]\geq y(t)$. because 0 ≤ *y*(*t*) ≤ 1, the equation can be simplified to:$\left[\left(-\Delta h+\frac{d}{2\beta })+\varphi gx(t\right)\right]-1\geq 0$,namely:$\left[\left(-\Delta h+\frac{d}{2\beta }\right)\right]-1\geq -\varphi gx(t)$. Because 0 ≤ *x*(*t*) ≤ 1,namely:$\left[\left(-\Delta h+\frac{d}{2\beta }\right)\right]-1\geq 0$.

### Appendix 2 Solution of continuous strategy set evolutionary game

During the game between the two parties, the Internet platform continuously adjusts its strategic choices based on maximizing strategic benefits. The conditions that guide the Internet platform to improve the level of fair competition are: $\frac{\partial EY\left(n\right)}{\partial n}>0$ is always true. By finding the first-order and second-order partial derivatives of Eq (15) with respect to *n*, we can get:

$$\frac{\partial EY\left(n\right)}{\partial n}=\int_{0}^{1}\left[-\Delta h+\frac{d}{\beta }(1-n)+m\varphi g\right]f\left(m\right)dm,$$ (25)

$$\frac{\partial^{2}EY\left(n\right)}{\partial n^{2}}=-\int_{0}^{1}\frac{d}{\beta }f\left(m\right)dm.$$ (26)

It can be seen from the value range set by the parameters that $\frac{\partial^{2}EY\left(n\right)}{\partial n^{2}}<0$, indicating that $\frac{\partial EY\left(n\right)}{\partial n}$ is a decreasing function on *n*ϵ[0,1], Get the minimum value when *n* = 1.


$${\left.{\frac{\partial EY\left(n\right)}{\partial n}}_{min}=\int_{0}^{1}\left[-\Delta h+m\varphi g\right]f\left(m\right)dm\right|}_{n=1}.$$ (27)

When ${\frac{\partial EY\left(n\right)}{\partial n}}_{min}\geq 0$, it means that *EY*(*n*) increases as the value of n increases, and the system eventually evolves toward fair competition. Since n obeys a uniform distribution in the interval [0,1], then $\int_{0}^{1}nf\left(n\right)dn=E\left(n\right)=\frac{1}{2}$. Therefore, the condition for Internet platform strategic choice to evolve toward fair competition is: $-\Delta h+\frac{\varphi g}{2}>0$.

### Appendix 3 Evolutionary conditions of equilibrium point (0,0) of continuous strategy set evolutionary game

The conditions for the game system to evolve to the negative state (0,0):

According to Eqs (18) and (19), $\frac{\partial EX\left(m\right)}{\partial m}$ obtains the maximum value at *m* = 0,

$${\left.{\frac{\partial EX\left(m\right)}{\partial m}}_{max}=\int_{0}^{1}\left[S-c_{0}-\frac{b\left(1-ep\right)}{\alpha }m+(1-n)qr\right]f\left(n\right)dn\right|}_{m=0}.$$ (28)

When ${\frac{\partial EX\left(m\right)}{\partial m}}_{max}<0$, it means that *EX*(*m*) decreases as the value of *m* increases, and the system eventually evolves in the direction of negative supervision. $S-c_{0}-+qr-\int_{0}^{1}qrnf\left(n\right)dn<0$, that is: $S-c_{0}+\frac{qr}{2}<0$. In the same way, the conditions for Internet platform strategic choice to evolve into unfair competition are: *$-\Delta h+\frac{d}{\beta }+\frac{\varphi g}{2}<0$*.

The expected benefits of the government department choosing regulatory intensity *m*:

$$EX\left(m\right)=\int_{0}^{1}\left[mS-{mc}_{0}-\frac{b\left(1-ep\right)}{2\alpha }m^{2}+nw-\left(1-n\right)v-(1-m)(1-n)qr\right]f\left(n\right)dn=-\frac{b\left(1-ep\right)}{2\alpha }m^{2}+\left(S-c_{0}+\frac{qr}{2}\right)m+\frac{w-v-qr}{2}.$$ (29)

The expected benefits of the Internet platform choosing fair competition level *n*:

$$EY(n)=\int_{0}^{1}\left[H+\left(1-n\right)\Delta h-\frac{d}{2\beta }{\left(1-n\right)}^{2}-m\left(1-n\right)\varphi g\right]f\left(m\right)dm=-\frac{d}{2\beta }n^{2}+(\frac{d}{\beta }+\frac{\varphi g}{2}-\Delta h)n+H+\Delta h-\frac{d}{2\beta }-\frac{\varphi g}{2}.$$ (30)
